# Comparison of microbiome isolated from the conjunctiva, contact lens and lens storage case of symptomatic and asymptomatic contact lens users

**Published:** 2019-10

**Authors:** L. Raksha, G.B. Shantala, Nagaraju Gangashettappa, R. Ambica, Deepa Sinha

**Affiliations:** 1 Department of Microbiology, Bangalore Medical College and Research Institute, Bangalore, Karnataka, India; 2 Department of Ophthalmology, Bangalore Medical College and Research Institute, Bangalore, Karnataka, India

**Keywords:** Microbial keratitis, Symptomatic contact lens users, Contact lens associated ocular microbiome, Dryness

## Abstract

**Background and Objectives:**

Contact lenses (CLs) are increasingly being used for cosmetic or therapeutic purposes. Lack of compliance and poor hygiene towards lens care is strongly associated with microbial contamination and has been proved to result in eye infections. The present study was done to compare the microbial flora between symptomatic and asymptomatic contact lens users. The study also attempts to analyze the contact lens hygiene practices of CL users.

**Materials and Methods:**

Six samples each were collected from both the eyes, CLs and lens cases of 40 CL users (n=240) divided into two groups based on symptoms present asasymptomatic CL users and symptomatic CL users. Organisms were identified using standard microbiological techniques.

**Results:**

The proportion GNB obtained in symptomatic CL users was significantly higher when compared to asymptomatic CL users (p-value= <0.003). In 56.2% eyes, the microbial flora of conjunctiva was similar to either the contact lens isolate/storage case. Enterococcal microbial keratitis was seen in one case.

**Conclusion:**

There was significant microbial contamination present in CL users despite compliance to contact lens hygiene practices. There were a significant number of bacteria (p-value <0.001) present which were resistant to ampicillin, amoxicillin-clavulanate, and cefotaxime in both the groups.

## INTRODUCTION

Normal conjunctival flora is either exogenous or endogenous in origin, which can be contracted from the environment, physical contact or unhygienic habits of people. One of the physical contacts is the use of contact lenses and also the unhygienic maintenance of the lenses (1). Many ocular infections occur when prosthetic devices come in contact with or are implanted in the eye such as Microbial keratitis (MK) (2), Contact lens-related acute red eye (3), corneal ulcer (4) and infiltrative keratitis (3). MK may result in vision loss as a consequence of corneal scarring (5).

Contact lenses (CLs) are increasingly being used for cosmetic or therapeutic purposes. CLs provide a wider field of vision, are not affected by weather conditions (fogging, getting steamed up) and provide a lesser distortion of images when compared to eye-glasses, hence are preferred. Lack of compliance and poor hygiene towards lens care is strongly associated with microbial contamination and has been proved to result in eye infections (6, 7). This may be due to pathogens introduced into the eye as a result of contact lens wear and corneal hypoxia, which interrupts the integrity of the epithelium and serves as an entry point for microorganisms (8). Contact lens storage case contamination has been shown to occur in both symptomatic and asymptomatic contact lens wearers even if good compliance with care regimes is practiced (9).

The incidence of corneal infections among contact lens users has not changed in the last 20 years as found by epidemiological studies (10, 11), though there are improvements in contact lens solutions and wearing habits. The sustained incidence of corneal infections shows that the microbes have enough potential to adapt to clinical modifications that interfere with their pathogenesis in causing the same. The identification of the infectious or non-infectious origin of contact lens-related keratitis and corneal ulcers is of paramount importance to effectively treat them. Inappropriate characterization and treatment of the causative microorganism may end in persistent infection, permanent damage to ocular tissues, diminished vision, and in worst cases removal of the infected tissue (12). There are studies which have shown that contact lens use alters ocular microbiome (13–15). Therefore, the present study was undertaken to compare the microbial flora between symptomatic and asymptomatic contact lens users and their anti-microbial susceptibility patterns. The study also attempts to analyze the contact lens hygiene practices of CL users.

## MATERIALS AND METHODS

### Study cases

The study was an observational study conducted in the Departments of Microbiology and Ophthalmology of a tertiary care setting in Bangalore, Karnataka, India. Institution ethical clearance was obtained. Informed written consent was obtained from those who volunteered to participate.

A total of 40 individuals in two groups of 20 each were included in the study.

Group 1: 20 asymptomatic contact lens users in the age group 18–35 years consisting of undergraduates and post-graduate medical students.

Group 2: 20 Symptomatic contact lens users in the age group 18–35 years consisting of undergraduates and post-graduates, studying at medical college. The study subjects were silicone hydrogel soft contact lens users (occasional or daily users).

Patients with any of the following symptoms were categorized as symptomatic contact lens users:

- Stinging, burning or itching (irritation) sensation in the eye
- Eye pain
- Abnormal feeling of something in the eye (foreign body, scratched area)
- Excessive watering (tearing) of the eyes
- Unusual eye secretions
- Redness of the eyes
- Reduced sharpness of vision (poor visual acuity) blurred vision, rainbows, or halos around objects,
- Sensitivity to light (photophobia)
- Dry eyes.

All the participants were examined by an ophthalmologist using a slitlamp. Individuals with ocular infections, co-existing ocular diseases, antibiotic use within one month and systemic diseases were excluded from the study. The basic demographic details of the patient, brief history and questionnaire regarding their contact lens hygiene practices were collected.

### Collection of conjunctival samples

One sample each from left and right eye was collected separately from 20 (n=40) asymptomatic CL users and 20 (n=40) symptomatic CL users by swabbing the lower conjunctival sac using sterile cotton swabs followed by transferring them immediately into BHI (Brain heart infusion) broth.

### Collection of samples from contact lenses and lens storage cases

Contact lenses were collected from individuals just as they were to be discarded (after a duration of one month in case of monthly disposable contact lenses and after 24 hours of use in case of the daily disposable lens) and placed in BHI (Brain heart infusion) broth. Samples from the contact lens storage case with the solution were collected by swabbing them with sterile cotton swabs.

Thus, 6 samples each was collected from contact lens users (Right and left Conjunctival sample, right and left contact lens, right and left contact lens storage case with a solution in it). A total of 240 samples in all were collected from both asymptomatic and symptomatic CL users.

### Processing of samples

After 24 hrs incubation at 37°C in BHI broth, the samples were sub-cultured onto Blood agar, Mac Conkey agar, and Sabouraud’s dextrose agar. The blood agar and Mac Conkey’s agar were incubated at 37°C for 24–48 h, while Sabouraud’s dextrose agar samples were incubated at 25°C and examined daily for the growth of fungi for three weeks before declaring them negative. Organisms grown were identified using standard microbiological techniques (16). The antimicrobial susceptibility test for bacterial isolates (*Staphylococcus aureus* and Gram-negative bacilli) was done by Kirby–Bauer disc diffusion method according to the Clinical and Laboratory Standards Institute (CLSI) guidelines 2017. The *Staphylococcus aureus* isolates were tested for susceptibility to antibiotics – penicillin (10 units), cefoxitin (30 μg), erythromycin (15 μg), clindamycin (2 μg), azithromycin (15 μg), tetracycline (30 μg), doxycycline (30 μg) and vancomycin (30 μg). Gram-negative bacilli isolates were tested for susceptibility to ampicillin (10 μg), amoxicillin (20 μg) + clavulanic acid (10 μg), azithromycin (15 μg), ciprofloxacin (5 μg), imipenem (10 μg), levofloxacin (μg), cefotaxime (30 μg) and ceftriaxone (30 μg).

The data obtained was in the form of percentages and were analyzed using appropriate statistical tests and represented using tables and bar graphs.

## RESULTS

### Asymptomatic contact lens users

**Fig. 1:**
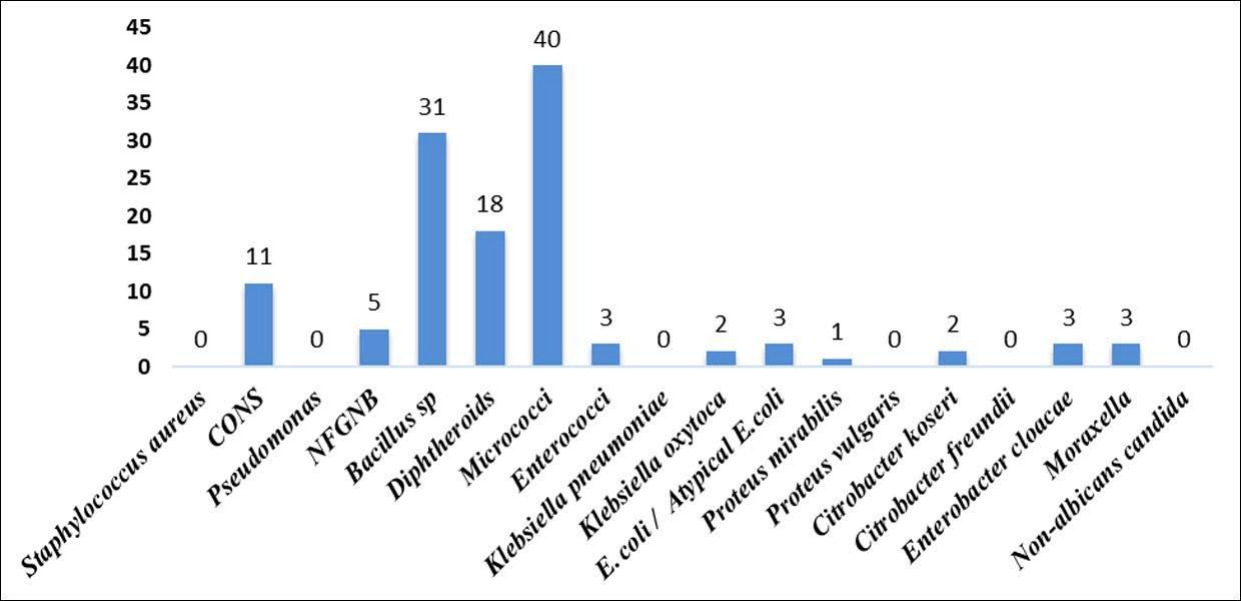
Frequency of microbial isolates from asymptomatic CL users

Of 120 contact lens samples obtained from the conjunctiva, contact lens and lens storage case of asymptomatic contact lens users 114 (95%) showed growth on Blood agar and MacConkey agar- 8 samples exhibited polymicrobial growth and 106 samples had monomicrobial growth. There was no fungal growth. In all n=122 bacterial isolates were obtained. The distribution of microbial isolates is depicted in Fig. 1.

### Symptomatic contact lens users

**Fig. 2:**
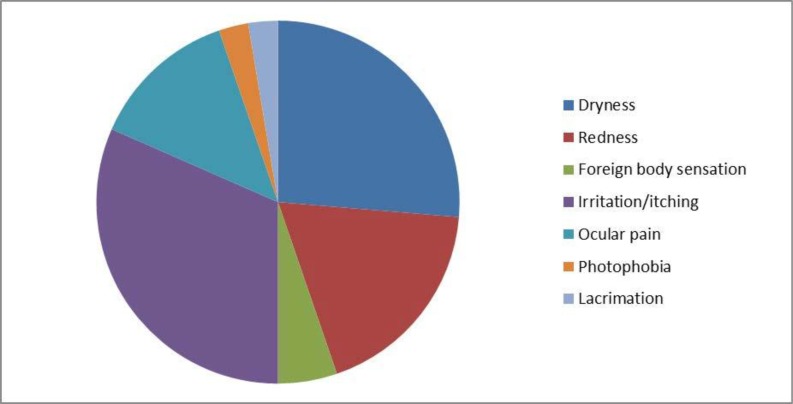
Symptoms reported by CL users

The symptoms/problems reported by contact lens users in our study are depicted in Fig. 2. More than one symptom was present in some of the study subjects.

On ocular examination, CLARE (Contact lens associated red eye) was present in 6 (30%) of CL users:

- Circumciliary congestion – 6 cases
- Central corneal edema- 1 case
- Conjunctival papillary reaction-6 cases

**Fig. 3:**
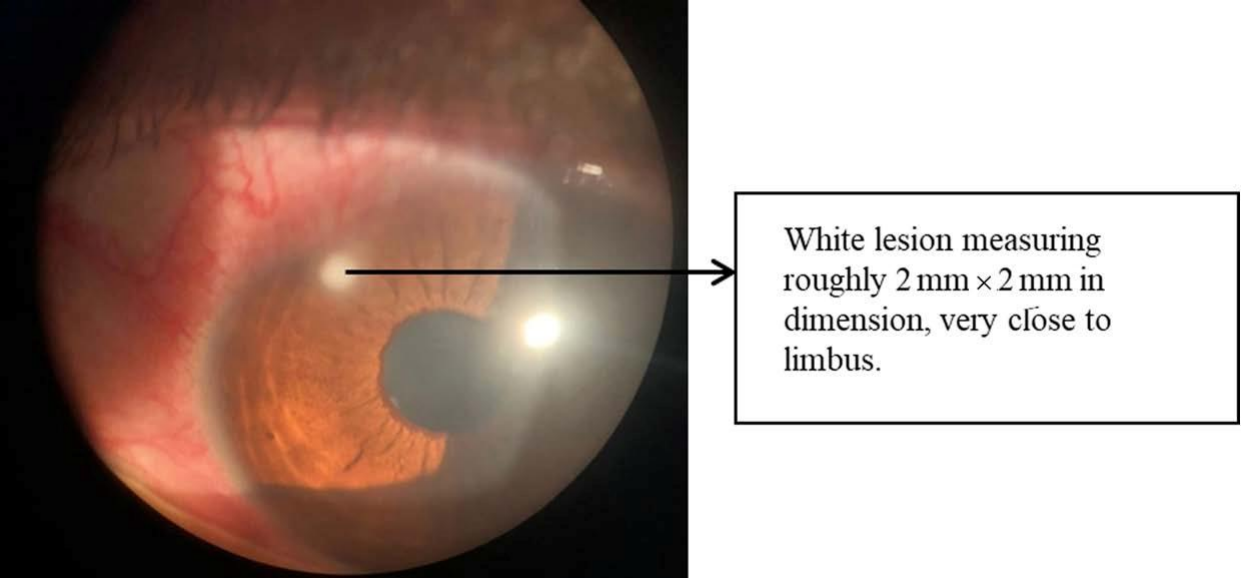
Lesion as seen on slit lamp examination

There was one case of keratitis seen-A 22 yr old female patient from Bangalore presented with complaints of itching and redness of the right eye. On examination, there was circumciliary congestion, central corneal edema, and the conjunctival papillary reaction seen. There was a white lesion present measuring roughly about 2 mm × 2 mm in dimension, very close to the limbus (Fig. 3). In this case, in addition to conjunctiva, contact lens and lens storage case samples of both the eyes, the corneal scrapings of the right eye were also taken. The conjunctiva, contact lens, lens case and corneal scraping sample of the right eye (the one with the chief complaint) showed growth of *Enterococcus* spp.

**Fig. 4:**
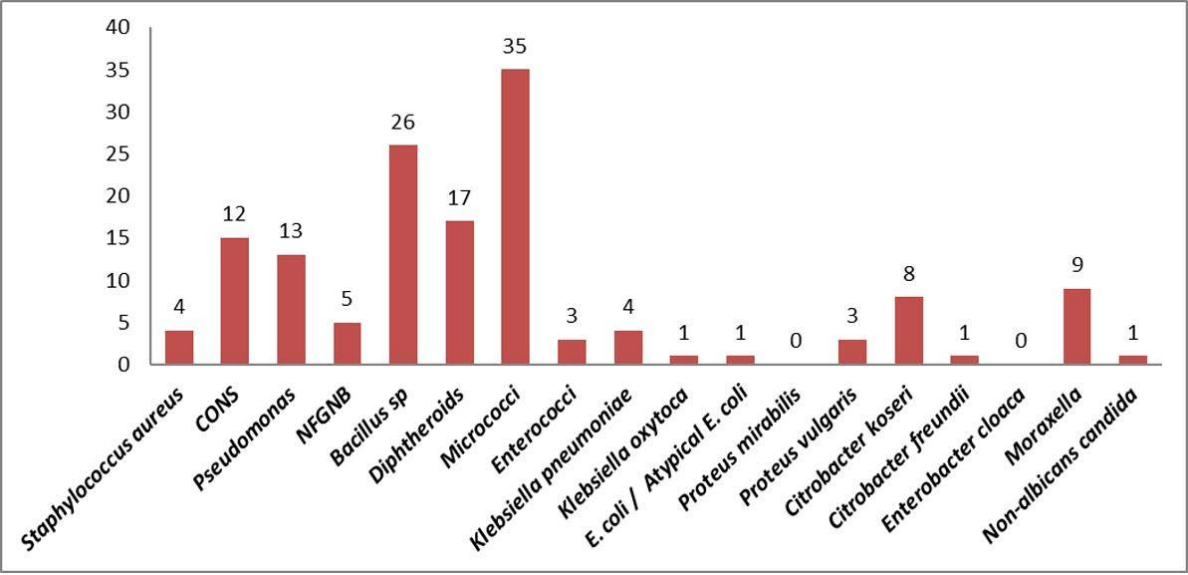
Frequency of microbial isolates from symptomatic users

Of 120 contact lens samples obtained from the conjunctiva, contact lens and lens storage case of symptomatic contact lens users, 120 (100%) of them yielded growth on Blood agar and MacConkey agar. One sample (from contact lens) showed growth on SDA agar- 19 samples exhibited polymicrobial growth and 109 samples had monomicrobial growth. The growth on SDA agar was identified to be Non-albicans candida and the person reported to have the symptom of dryness. In all n=143 bacterial isolates were obtained. The distribution of microbial isolates is depicted in Fig. 4. The methicillin-sensitive *Staphylococcus aureus* (MSSA) isolates were resistant to penicillin, erythromycin, clindamycin, and azithromycin but sensitive to cefoxitin, tetracycline, doxycycline, and vancomycin. Other organisms obtained were *Moraxella*, NFGNB, *Klebsiella pneumoniae*, *Enterococcus, Enterobacter, Citrobacter koseri, Proteus vulgaris, E. coli, Klebsiella oxytoca* and *Citrobacter freundii*.

### Antimicrobial susceptibility testing

There were 65 Gram-negative Bacilli (GNB) obtained (symptomatic and asymptomatic contact lens users).

Of them 49 were ampicillin resistant, 42 were amoxicillin-clavulanate resistant and 18 were cefotaxime resistant (p-value < 0.001) (Table 1) and all were sensitive to ceftriaxone, imipenem, levofloxacin, amikacin, and ciprofloxacin.

**Table 1:** Chi-square analysis of antimicrobial susceptibility pattern

	**Resistant**	**Sensitive**
Ampicillin	49	16
Amoxicillin Clavulanate	42	23
Cefotaxime	18	47

P<0.001^**^, Significant, Chi-Square Test

### Comparison between asymptomatic and symptomatic CL users

**Fig. 5:**
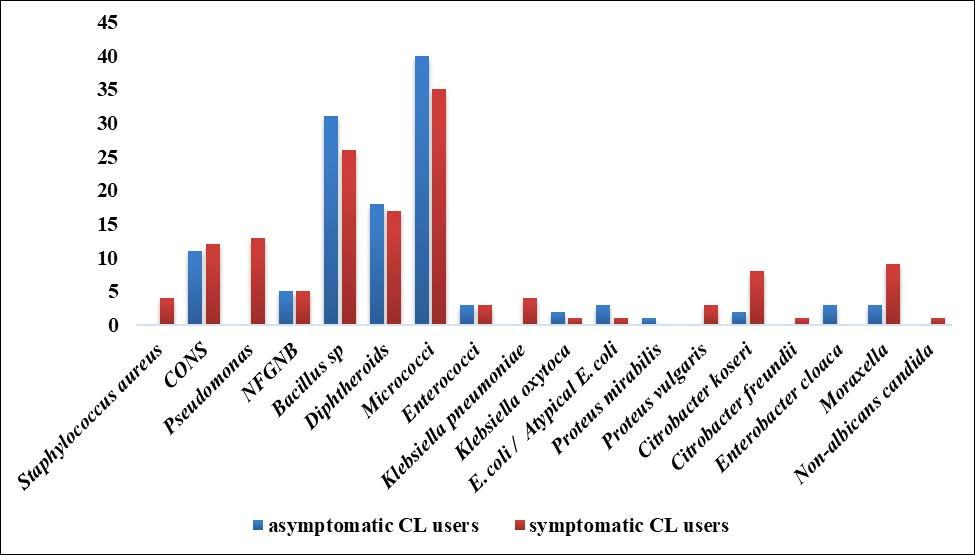
Comparison of microbial isolates from asymptomatic and symptomatic CL users

*Pseudomonas, Staphylococcus aureus, Klebsiella pneumonia, Proteus vulgaris*, Non-albicans *Candida* isolates were obtained only from symptomatic CL users and none from asymptomatic CL users (Fig. 5). The proportion of GNB obtained in symptomatic CL users was significantly higher when compared with asymptomatic CL users (p-value=0.003) (Table 2).

**Table 2:** Chi-square analysis of GNB between asymptomatic CL users and symptomatic CL users

**Gram-negative bacteria**	**Asymptomatic CL users**	**Symptomatic CL users**
No of isolates present	19 (15.6%)	45 (31.5%)
No of isolates absent	103 (84.4%)	98 (68.5%)

P=0.003^**^, Significant, Chi-Square Test

### Analysis of contact lens hygiene practices

All the individuals who participated in the study wore contact lenses for optical indications: refractive error (myopia). There was little or no difference in contact lens hygiene practices amongst asymptomatic CL users and symptomatic CL users. Though there was good compliance to contact lens care practices, there was microbial contamination seen with pathogens, which was higher in symptomatic cases (Table 3).

**Table 3:** Analysis of contact lens hygiene practices

	**Frequency**
**The frequency of lens usage**	
Daily	29 (72.5%)
Occasional	11 (27.5%)
**Duration of use**	
>8 hrs	19 (47.5%)
<8hrs	21 (52.5%)
**Type of contact lens**	
Daily wear	1 (2%)
Monthly	34 (85%)
Quarterly	3 (7%)
Yearly	2 (5%)
**Contact lens use while swim/shower**	
Yes	0
No	40 (100%)
**Contact lens use while sleeping**	
Yes	0
No	40 (100%)
**Use of water in cleaning contact lens and accessories**	
Yes	0
No	40 (100%)
**Washing hands with soap and water before touching contact lens**	
Yes	38 (95%)
No	2 (5%)
**Rub and rinse of contact lens (following steps of lens cleaning protocol)**	
Yes	40 (100%)
No	0
**Frequency of cleaning the contact lens**	
Everyday	29 (72.5%)
Weekly	11 (27.5%)
**Storage of lens**	
**Lens case**	40 (100%)
**Other**	0

## DISCUSSION

Microbial isolates from the conjunctiva, contact lens and its accessories of contact lens users were studied in two groups-asymptomatic and symptomatic CL users. When results are combined, the overall rate of microbial contamination of samples obtained from the conjunctiva of contact lens users, contact lens and accessories was 97.5 per cent (234/240) which differs from studies conducted by Lipener et al. (17) (86.6%) and *Emina* et al. (18) (70.27%). The sampling technique for contact lens used in our study where the whole contact lens was transferred into the broth after a certain period of use is different from other studies where swabbing of contact lens is done. This kind of sampling technique is unique and helped us in getting a high percentage of growth (97.5%). Of the 80 eye samples taken (asymptomatic and symptomatic CL users), in 45 (45/80=56.2%) eyes, the microbial flora of conjunctiva was similar to either the contact lens isolate/storage case which supports the statement that the pathogens in the conjunctiva are acquired from the contact lens and its accessories. Further studies to explore the effectiveness of the lens care antiseptic solution in preventing the same is required.

There were a significant number of bacteria (p-value <0.001) present which were resistant to ampicillin, amoxicillin-clavulanate, and cefotaxime in both asymptomatic and symptomatic CL users. Hence, the present study can be used as a guide to formulate antibiotic policy for empirical treatment or prophylaxis in CL users.

The commonest isolate obtained from the asymptomatic contact lens users group in our study is micrococci (32.7%) followed by *Bacillus* species (25.4%), diphtheroids (14.7%) and CONS (9%). which differs from other studies where the highest obtained microbial isolate is either *Pseudomonas* (19) or CONS (1, 20). The proportion of organisms - *Citrobacter koseri, Moraxella,* Enterococci, NF-GNB, *Proteus vulgaris, Enterobacter cloacae, E. coli, Proteus mirabilis* reported in our study is higher compared with studies done by Rahim N et al. (1) and *Lipener* C et al. (17).

To the best of our knowledge, the present study is the first in literature done comparing the microbial isolates from asymptomatic and symptomatic CL users. The study is also one of its kinds in specifically categorizing symptomatic CL users and analyzing their microbial flora. There was a notable difference in microbial isolates obtained from the same. Symptomatic CL users (15.8%) showed a higher percentage of polymicrobial growth compared with asymptomatic CL users (7%). *Pseudomonas, Staphylococcus aureus, Klebsiella pneumoniae, Proteus vulgaris*, Non-albicans *Candida* isolates were obtained only from symptomatic CL users and none from asymptomatic CL users. The proportion of GNB obtained from symptomatic CL users was significantly higher when compared with asymptomatic CL users (p-value= <0.003). There were 2 MRSA isolates obtained from symptomatic CL users in our study which may be attributed to the exposure to the hospital environment of our study population. Dryness and itching/irritation of the eye were the commonest complaint among symptomatic contact lens users. There was one case of MK secondary to CL wear and enterococci were isolated from the conjunctiva, contact lens, and storage case samples of the patient. *Enterococcus* sp, though rare has still been implicated in causing MK as reported by *Rau G* et al. (21). Hence, the present study shows that the reason for discomfort/symptoms in CL users may be due to the aforementioned microbial isolates which are not present in asymptomatic CL users. However, further studies are required to prove the association of microbial isolates with presenting signs and symptoms in symptomatic CL users. When the contact lens hygiene practices were analyzed, there was no significant difference between asymptomatic and symptomatic CL users. In the present study, despite reportedly good compliance with hygiene care practices and the study subjects consisting of educated individuals there was bacterial contamination present (97.5%) and the finding is similar to study conducted by Stapleton et al. (9).

The limitation of the study: a) Follow up of the symptomatic CL users was not done. b) The study population included the only undergraduate and postgraduate medical student. Sampling of the wider population consisting of people from the non-healthcare background is required to reflect the exact microbiome in CL users.

## CONCLUSION

There was significant microbial contamination present in CL users despite compliance to contact lens hygiene practices and a significant number of isolates were resistant to amoxicillin clavulanate, ampicillin, and cefotaxime. There was a remarkable difference in the isolates obtained from asymptomatic and symptomatic contact lens users where *Pseudomonas, Staphylococcus aureus, Klebsiella pneumoniae, Proteus vulgaris,* Non-albicans *Candida* were found only in the contact lens group. The unique kind of sampling technique (transferring the CL lens directly into the broth after a month of use just when they were to be discarded) used in our study resulted in a high percentage of growth (97.5%). The present study explored the various organisms which may result in ocular infections. Thus, there is a constant change in the trend of pathogens in ocular infections and similar studies at regular intervals of time are necessary to design new antibiotic policies targeting the same.
